# Ultrasound Can Detect Macroscopically Undetectable Changes in Osteoarthritis Reflecting the Superficial Histological and Biochemical Degeneration: Ex Vivo Study of Rabbit and Human Cartilage

**DOI:** 10.1371/journal.pone.0089484

**Published:** 2014-02-21

**Authors:** Kohei Nishitani, Masahiko Kobayashi, Hiroshi Kuroki, Koji Mori, Takaaki Shirai, Tsuyoshi Satake, Shinnichiro Nakamura, Ryuzo Arai, Yasuaki Nakagawa, Takashi Nakamura, Shuichi Matsuda

**Affiliations:** 1 Department of Orthopaedic Surgery, Graduate School of Medicine, Kyoto University, Kyoto, Japan; 2 Department of Physical Therapy, Human Health Sciences, Graduate School of Medicine, Kyoto University, Kyoto, Japan; 3 Department of Applied Medical Engineering Science, Graduate School of Medicine, Yamaguchi University, Ube, Japan; 4 Department of Orthopaedic surgery, National Hospital Organization Kyoto Medical Center, Kyoto, Japan; SERGAS, Santiago University Clinical Hospital, IDIS Research Laboratory 9, NEIRID Lab, Spain

## Abstract

Recognizing subtle cartilage changes in the preclinical stage of osteoarthritis (OA) is essential for early diagnosis. To this end, the ability of the ultrasound signal intensity to detect macroscopically undetectable cartilage change was investigated. In this study, cartilage of rabbit OA model and human OA samples was examined by macroscopic evaluation, ultrasound signal intensity, histology with Mankin scores, and Fourier transform infrared imaging (FTIRI) analysis. Rabbit OA was induced by anterior cruciate ligament transection and evaluated at 1, 2, 4 and 12 weeks. Twenty human samples were harvested during total knee arthroplasty from OA patients who had macroscopically normal human cartilage (ICRS grade 0) on the lateral femoral condyle. In the animal study, there was no macroscopic OA change at 2 weeks, but histology detected degenerative changes at this time point. Ultrasound signal intensity also detected degeneration at 2 weeks. In human samples, all samples were obtained from macroscopically intact site, however nearly normal (0**≤** Mankin score <2), early OA (2≤ Mankin score <6), and moderate OA (6≤ Mankin score <10) samples were actually intermixed. Ultrasound signal intensity was significantly different among these 3 stages and was well correlated with Mankin scores (R = −0.80) and FTIR parameters related to collagen and proteoglycan content in superficial zone. In conclusion, ultrasound can detect microscopic cartilage deterioration when such changes do not exist macroscopically, reflecting superficial histological and biochemical changes.

## Introduction

Osteoarthritis (OA) is a slow progressive degenerative joint disease and is a leading cause of impaired mobility in the elderly [1]. Although it is clear that the early diagnosis of OA is important, there is no established method to detect very early or subtle changes in the OA cartilage. Clinically, plain radiographs are still the gold standard for staging OA. However, in the early stage of OA, little or no changes are apparent in plain radiographs [1]. Magnetic resonance imaging is more powerful tool than plain radiographs for the early diagnosis of OA, however cost and availability still remain significant hurdles [2]. It is useful to develop methods to detect such early changes in the cartilage. Moreover, basic studies detecting subtle changes in the cartilage matrix that accompany the progression of OA are pivotal in improving diagnostic methods.

Quantitative ultrasound is a candidate method for detecting subtle changes in the cartilage [3]. Various unique ultrasound devices have been used to investigate cartilage and the potential of ultrasound to evaluate subtle changes in the cartilage is promising [3], [4]. We developed our ultrasound noncontact method to evaluate the cartilage in an animal model. More recently, an ultrasound noncontact arthroscopy probe was used to evaluate knee and elbow cartilage during surgery [5], [6], [7]. Our recent report shows that the ultrasound signal intensity (US signal intensity) is useful for differentiating normal (ICRS grade 0) from slightly degenerated cartilage (ICRS grade 1) [7]. We believe that this ultrasonic noncontact probe is useful in evaluating subtle changes in the cartilage.

Our hypothesis is that the US signal intensity is sufficiently sensitive to detect the microscopic degeneration of articular cartilage. Therefore, we determined whether ultrasound can detect macroscopically undetectable histological changes in OA by using a rabbit OA model. We also used human macroscopically intact cartilage samples (ICRS grade 0) to clarify the ability of ultrasound to differentiate cartilage that has such intact surface.

## Materials and Methods

### Ethics Statement

All animal studies were conducted in accordance with principles by Kyoto University Committee of Animal Resources, based on International Guiding Principles for Biomedical Research Involving Animals. All procedures for this study were approved by Kyoto University Committee of Animal Resources (Permit Number: Med Kyo 10184). For all human species, ethical approval for this study was granted by the ethics committee of Kyoto University Graduate School and Faculty of Medicine. Written informed consent was provided and obtained from all study participants.

### Animal Samples

Eighteen skeletally matured female Japanese white rabbits (weight, 4.0–4.5 kg) were used. Two rabbits (4 knees) were allocated to 0-week control, and 4 were randomly allocated into 4 groups that were examined at 1, 2, 4, or 12 weeks after surgery. They were individually housed at 22°C and 50% humidity with a 14/10-h light/dark cycle and had free access to food and water. For operations, intravenous pentobarbital sodium (25 mg/kg) was used to induce and maintain general anesthesia. An intraarticular injection (3 mL) of 1% lidocaine to each knee was used and a medial parapatellar incision was made to expose both knee joints. Bilateral anterior cruciate ligaments were exposed, and left anterior cruciate ligament transection (ACLT) was carefully performed. The joint capsule and skin incision were closed. The rabbits were allowed full weight-bearing postoperatively. The animals were sacrificed by an intravenous injection of pentobarbital sodium (100 mg/kg) and were macroscopically evaluated by two orthopaedic surgeons. In brief, cartilage changes were graded on a scale from 0 to 5 (0, intact surface normal in appearance; 1, minimal fibrillation; 2, overt fibrillation, distinguished surface irregularity, or cracks; 3, erosion from 0 to 2 mm; 4, erosion from 2 to 5 mm; 5, erosion >5 mm) [8]. The cartilage samples were stored at −20°C before use.

### Human Samples

Human articular cartilage samples were obtained from a series of total knee arthroplasties of OA. Patients were diagnosed as OA according to the criteria of the American College of Rheumatology [9]. The KL grading system was used to score knees [10]. The cartilage of the weight-bearing area of the lateral femoral condyle was graded, using the International Cartilage Repair Society (ICRS) grading system by two experienced orthopaedic surgeons [11]. Patients were included in the study only when there was an ICRS grade 0 lesion in the weight-bearing area of the lateral femoral condyle. Eventually, three males and 17 females (mean age, 73.9 years; range, 55–82) (10 patients with KL grade 3 and 10 patients with KL grade 4) were involved in this study. One cylindrical osteochondral plug (diameter, 6 mm) for each patient was harvested from the center of the ICRS grade 0 lesion. The cartilage samples were stored at −20°C before use.

### Ultrasound Evaluation

**Figure 1:**
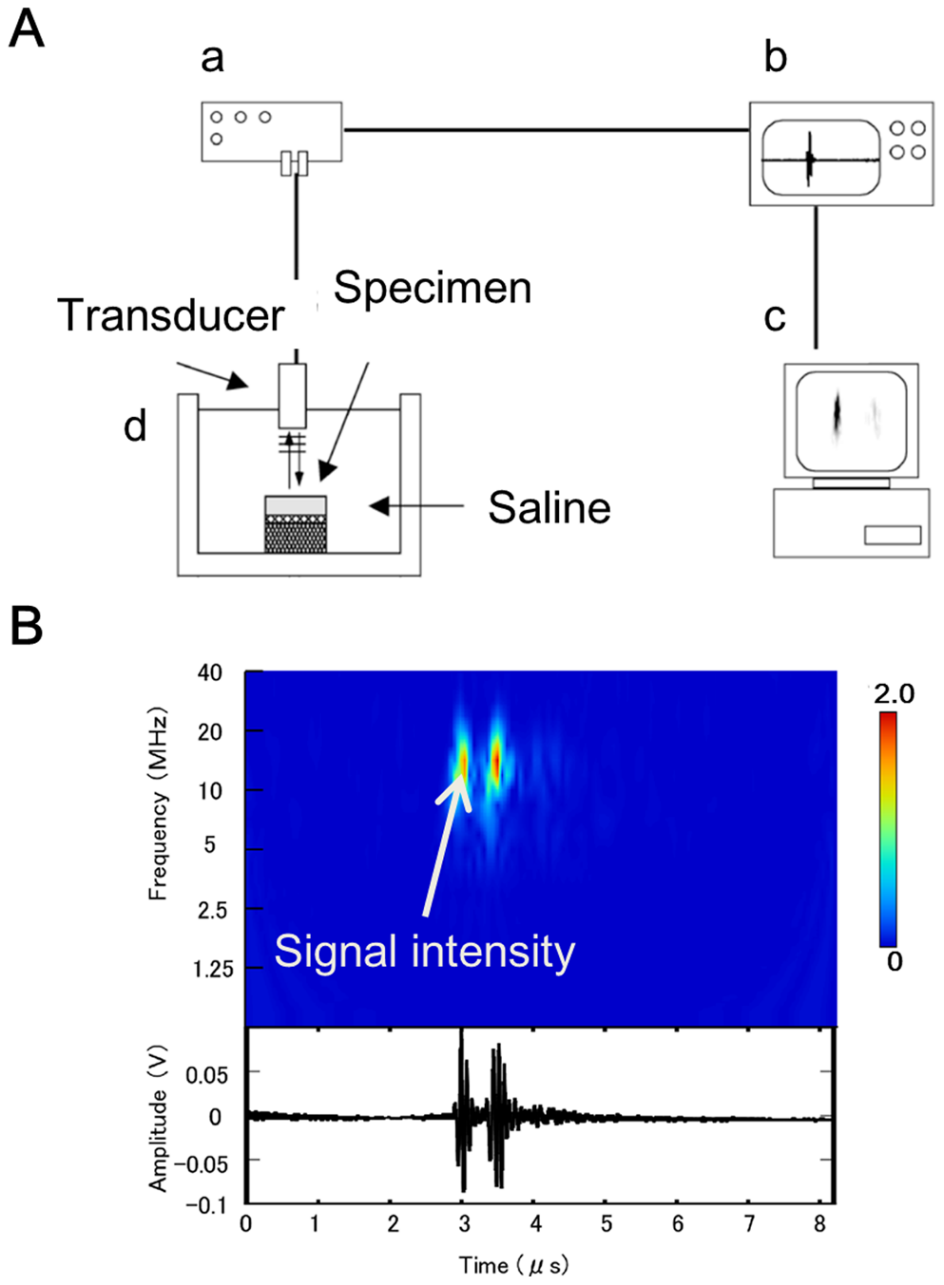
A scheme of the ultrasound measurement system. A) The ultrasound measurement system consists of a transducer, a pulser/receiver (a), a digital oscilloscope (b), and a personal computer (c), and saline bath and probe (d). B) typical A-mode echogram (lower) and its wavelet map (upper) of the cartilage. Each diagram has 2 peaks. The left one is a reflex echo from the surface, and the right one is from the subchondral bone. The wavelet map provides comprehensive information on the transient distribution of the intensity and frequency of an echo wave. US signal intensity is shown by graduation on the wavelet map.

The ultrasonic measurement system with noncontact probe has been described previously and provides a quantitative assessment of tissues properties [5], [12], [13]. In brief, the transducer is 3 mm in diameter, and the center frequency of the ultrasonic signal is 10 MHz (Figure 1A). While examining cartilage, 2 large-amplitude groups of reflected waves are observed: one is from the cartilage surface and the other is from the subchondral bone (Figure 1B). The maximum magnitude of the wave reflected from the articular cartilage surface on the wavelet map was defined as US signal intensity. Each animal specimen was examined at 3 different sites: at the center, and 5 mm anterior and posterior to the center of the weight-bearing area. Each measurement was performed twice, and data from the 3 points were averaged. Each human specimen was examined at the center of the cylindrical osteochondral plug; each measurement was performed twice, and the average value was used.

### Histological Analysis

Samples were fixed in 4% paraformaldehyde. Animal and Human samples were decalcified with 0.25 mol/L ethylenediaminetetraacetic acid in phosphate-buffered saline solution (pH 7.4) and Morse’s solution (10% sodium citrate and 22.5% formic acid), respectively. Sagittal sections (6-µm thick) were cut, stained with hematoxylin and eosin (HE) and safranin O/fast green (safranin/O). Histological scoring of the cartilage was performed by two blinded investigators and averaged by using the Mankin scoring system with the following 4 categories: cartilage structure (6 points), cartilage cells (3 points), staining (4 points), and tidemark integrity (2 points); normal and severely degenerated cartilage scoring 0 and 14, respectively [14]. Referring to the classification of Mankin and his colleagues, human specimens were classified into 4 stages based on their Mankin scores: nearly normal (0**≤** Mankin score <2), early OA (2**≤** Mankin score <6), moderate OA (6**≤** Mankin score <10), and late OA (10**≤** Mankin score **≤**14) [15], [16].

### FTIRI Evaluation

FTIRI was used to determine the spatial distribution of proteoglycan and collagen. The paraffin sections were mounted on metal plates and deparaffinized. A Fourier transform infrared spectrometer (FT-IR-460 PLUS, JASCO, Tokyo) coupled to a microscope (Intron-IRT-30, JASCO, Tokyo) was used for data acquisition. Spatial pixel size was 20×20 µm. Spectral resolution was set to 4 cm^−1^ wavenumber, and a spectral region of 2000∼670 cm^−1^ was collected. An integrated absorbance area of the carbohydrate region (1150–950 cm^−1^) and that under the amide I peak (1710–1595 cm^−1^) were defined as the proteoglycan and collagen contents, respectively [17], [18]. Two slices were evaluated for each cartilage and the averaged collagen and proteoglycan contents of the superficial zone and whole cartilage were used for quantitative analysis.

### Statistical Analysis

All values are means ± standard deviation. Student’s *t*-test was used for comparing sham and ACLT sides in the animal study. One-way ANOVA with a post hoc comparison was used to analyze differences among OA stages in the human study. Pearson’s linear correlation coefficient was used to determine correlations between the ultrasound and FTIRI parameters. Spearman’s correlation coefficient was used to determine correlations between the ultrasound parameter and Mankin scores. Statistical significance was set at P<0.05.

## Results

### Animal Specimens

**Figure 2:**
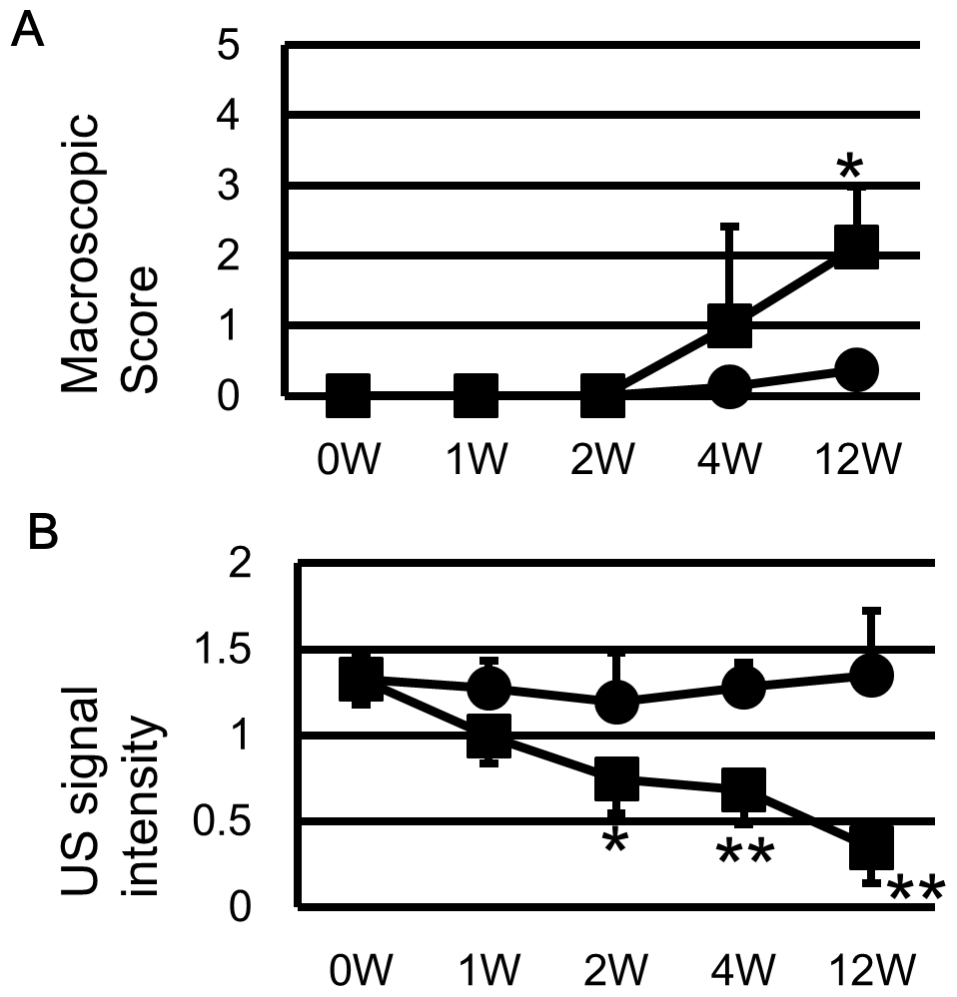
US signal intensity detected the deterioration of rabbit cartilages ahead of the macroscopic change. A) Macroscopic score of sham and ACLT side showed significant difference only at 12 weeks. B) US signal intensity of ACLT side decreased overtime and had significant difference to the sham side as early as 2 weeks. *: p<0.05 to sham side. **: p<0.01 to sham side.

The sham side of the LFC showed little OA-like changes throughout the experimental period. At 1 or 2 weeks, there was no visible change at the LFC in the ACLT side. At 4 weeks, some rabbits exhibited slight LFC fibrillation in ACLT side. At 12 weeks, most rabbits exhibited fibrillation at the LFC of ACLT side. The macroscopic scores of the LFC were significantly higher only at 12 weeks (Figure 2A).

### Ultrasound Evaluation (Figure 2B)

The US signal intensity of the ACLT side decreased with time. At 2 weeks, the US signal intensity of ACLT (0.74±0.20) was significantly lower than that of sham (1.19±0.29). At 4 and 12 weeks, the US signal intensity of the LFC (0.68±0.20 and 0.34±0.21, respectively) decreased further and was significantly lower than sham side (1.28±0.14 and 1.35±0.37, respectively).

### Histological Evaluation (Figure 3A)

**Figure 3:**
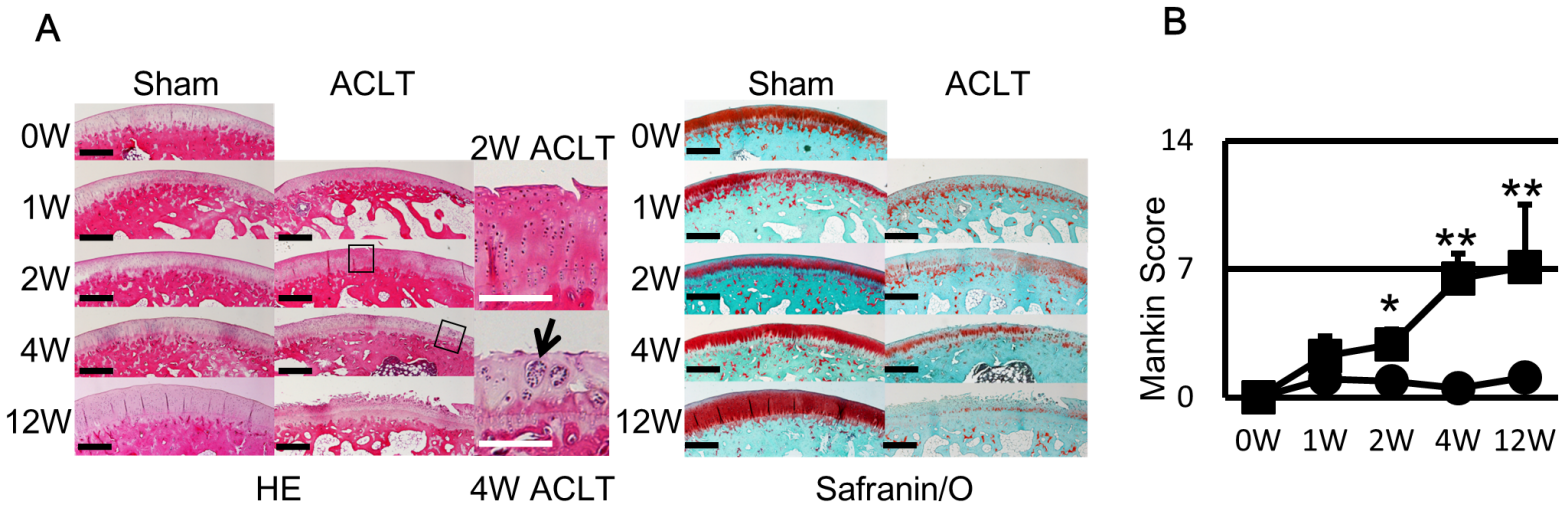
Histological sections and Mankin score of rabbit cartilages also showed OA change at 2 weeks. A) OA change such as fibrillation of the surface and decrease of safranin/O stainingwas detected from 2 weeks and deteriorated overtime. At 12 weeks, most of samples clearly showed OA change with fissures and further decrease of safranin/O staining. In HE, Black boxes of 2W and 4W ACLT side are showed in right column. Black arrow indicates the cloning of chondrocytes. D) Mankin score of ACLT side increased overtime and there was significant difference after 2 weeks, like US signal intensity. Black bar in histology indicates 100 µm and white bar indicates 50 µm. *: p<0.05 to sham side. **: p<0.01 to sham side.

At one week, both the ACLT and sham sides showed reduced safranin/O staining. At 2 weeks, although the safranin/O staining of the sham side looked restored, the surfaces of the ACLT group appeared crackled and the safranin/O staining was not restored, and started to show OA-like changes. At 4 weeks, the safranin/O staining of the sham side was completely restored. The ACLT side exhibited fibrillation of the cartilage surface and chondrocyte cloning. At 12 weeks, the sham side appeared normal, whereas the ACLT side exhibited progression of OA-like changes, including fibrillation increase, chondrocyte cloning, and decrease in safranin/O staining. The Mankin scores (Figure 3B) of the ACLT side continuously increased and the Mankin scores of both sides were significantly different at 2, 4 and 12 weeks.

### Human Specimens

Two specimens were excluded due to unsuccessful decalcification process of the paraffin sections. Mankin scores ranged widely (0 to 8), although all samples were obtained from ICRS grade 0 sites. The samples were classified into the following stages: nearly normal (3 samples), early OA (12 samples), and moderate OA (3 samples). There were no samples which were classified as late stage.

### Histological Evaluation (Figure 4)

Samples in the nearly normal stage had smooth surface, dense safranin/O staining, and columnar chondrocyte arrangements. FTIRI mapping showed Amide I rich area in superficial zone and Carbohydrate region rich area along with Safranin/O staining. In the early OA stage, samples showed slight fibrillation and reduced safranin/O staining and its amide I and carbohydrate region rich area decreased. In the moderate OA stage, fibrillation became obvious and chondrocyte arrangement became random, safranin/O staining decreased, and the amide I or carbohydrate rich area was not seen.

**Figure 4:**
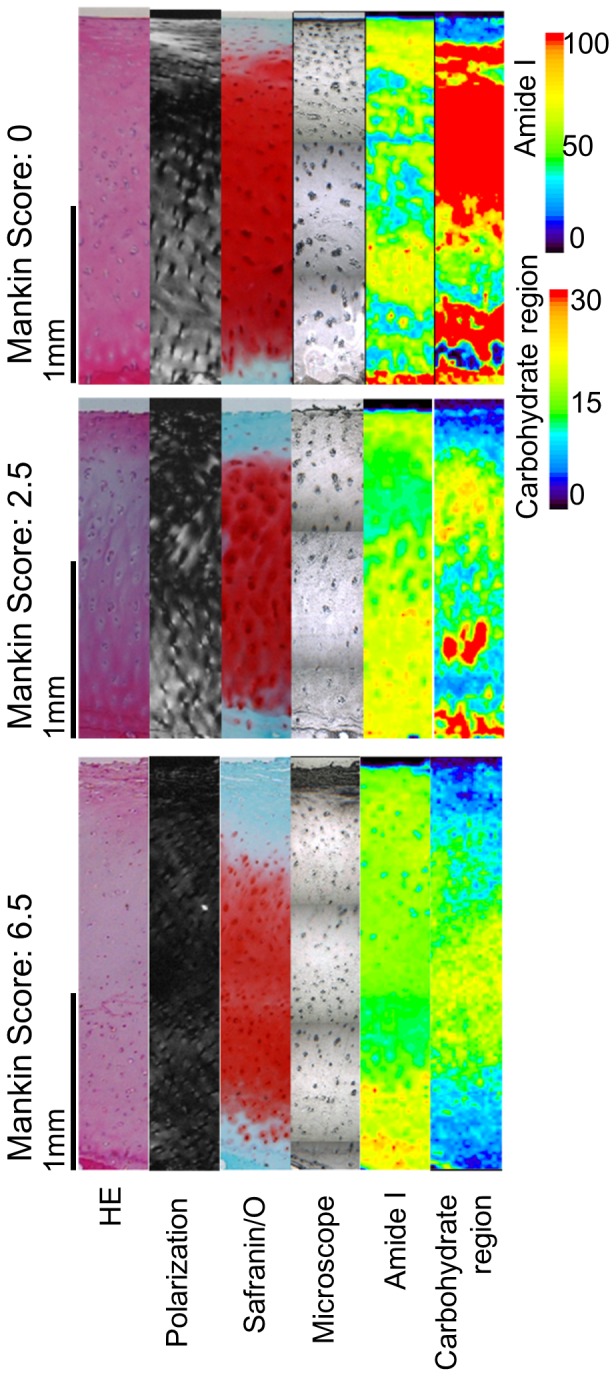
Though gross surface appearance was similar, histology section showed variety of OA stages. Histological section of the representative samples of each stage. HE, poralized microscope of HE, Safranin/O, Microscope for FTIR region of interest, Amide I mapping of FTIR and Carbohydrate region mapping of FTIR are shown. Upper panel is patient 1, middle is patient 2 and lower is patient 7. Black bar indicate 1 mm. Nearly normal cartilage showed a smooth surface, superficial collagen fiber network parallel to the surface, dense Safranin/O staining, Amid I rich area in superficial layer and Carbohydrate region rich areas in whole layer. As cartilage degeneration, these findings disappear or decrease.

### Ultrasound Evaluation (Figure 5)

US signal intensity of the nearly normal stage was significantly higher than early or moderate stage, and US signal intensity of early stage was significantly higher than moderate stage.

**Figure 5:**
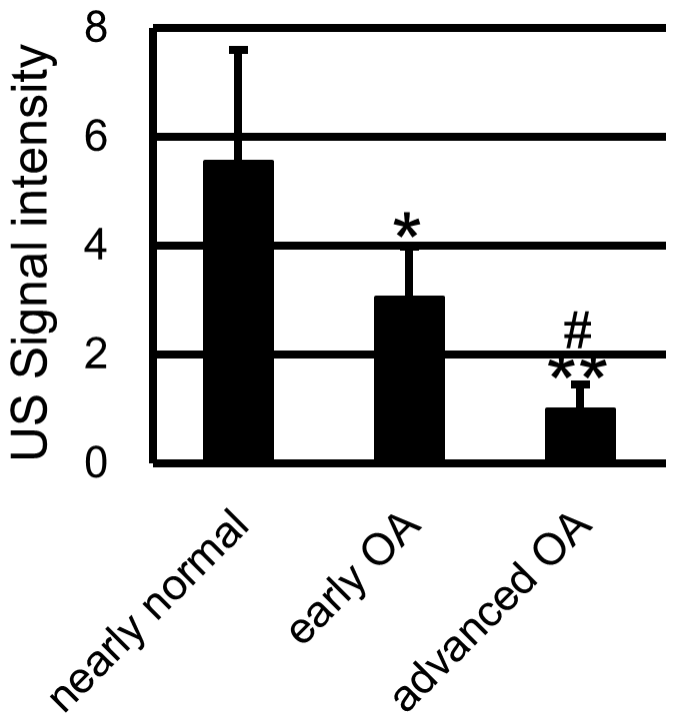
US signal intensity of macroscopically intact human specimens was considerably different among 3 histological stages. US signal intensity of the nearly normal cartilage was significantly higher than that of early OA or moderate OA cartilage. There was also difference between early OA and moderate OA. *: p<0.05 to nearly normal stage. **: p<0.01 to nearly normal stage. #: p<0.05 to early OA stage. ##: p<0.01 to early OA stage.

### Correlation with Ultrasound Parameter

US signal intensity was significantly correlated with Mankin scores and its sub categories, including structure, cells, and safranin/O staining, but not tidemark (Table 1). There was a strong correlation between US signal intensity and the amide I value of the superficial zone. On the other hand, there was no significant correlation between US signal intensity and the amide I values of the whole zone (Table 2). Meanwhile, there was a significant correlation between US signal intensity correlated to carbohydrate region values of both the whole zone and the superficial zone, and correlation with the superficial zone was greater than that of whole zone (Table 2).

**Table 1:** Correlation of Mankin Score and its subcategories with US signal intensity.

	Correlation with US signal Intensity	
	correlation coefficient	P value
Mankin Score	−0.80	<0.001
I. structure	−0.72	0.002
II. cells	−0.67	0.004
III. Safranin/O staining	−0.58	0.01
IV. Tidemark	−0.42	0.06

**Table 2:** Correlation of Amide I and Carbohydrate region value with US signal intensity.

		Correlation with US signal Intensity	
		correlation coefficient	P value
Amide I value	superficial zone	0.82	<0.001
	whole zone	0.06	0.80
Carbohydrate region value	superficial zone	0.74	<0.001
	whole zone	0.61	0.006

## Discussion

The US signal intensity was able to detect macroscopically undetectable OA changes in both animal and human samples. In the animal part, there were no macroscopic changes in the ACLT side at 2 weeks; however, the US signal intensity of the ACLT side was already lower than that of the sham side. The histological Mankin scores also showed degenerative changes in the sham side at 2 weeks. Although all the human samples appeared intact, some samples were histologically degenerated. There was a significant difference among the US signal intensity of nearly normal, early OA, and moderate OA samples.

In the animal study, the US signal intensity detected cartilage deterioration when changes could only be identified histologically. OA changes were not macroscopically identified until 4 weeks after ACLT. However, upon histological evaluation, changes in OA, such as tiny crackle on the cartilage or decreased safranin/O staining, were observed at 2 weeks. Thus, ultrasound differentiated changes at this early stage.

In the human samples, although all cartilage samples were obtained from macroscopically intact areas (ICRS grade 0), Mankin scores varied widely. Three moderate OA samples out of 20 were actually diagnosed as normal by a trained orthopaedic surgeon. In clinical situations, orthopaedic surgeons mainly diagnose cartilage changes macroscopically. Thus, evaluating articular cartilage solely based on external findings is unreliable and more objective and distinct decision standards are required. We think US signal intensity with noncontact probe is possible candidate for objective evaluation of cartilage quality.

In human species, the correlation between US signal intensity and the subcategory of Mankin score showed the strongest correlation with structure which was largely from the superficial layer information but no correlation with tidemark which was from the information of deep region. US signal intensity was also strongly correlated with the amide I value of the superficial zone, but not with the whole zone. In terms of the correlation between US signal intensity and carbohydrate region value, correlation of superficial zone was greater than that of whole zone. The correlation of ultrasound reflection to collagen or proteoglycan content is previously reported [19], [20], [21]. Moreover, we and other groups reported that the reflected ultrasound waves provide superficial information of the cartilage [19], [22], [23]. The numerical analysis of ultrasonic propagation in articular cartilage from another study of our group suggests that the collagen content from the surface to one wave length (1600[m/s]/10 MHz = 0.16 mm) is correlated with US signal intensity [24]. Since the depth of the superficial zone was ∼100 µm, the information of the superficial zone was included in the depth of ultrasonic propagation. The depth of rabbit cartilage was 100–200 µm, which was adequate for our evaluation device. Thus, these correlations with the superficial zone information shows that US signal intensity reflect the cartilage degeneration of superficial zone.

There are some disagreement concerning the use of US signal intensity [25], [26], as US signal intensity cannot directly measure any intrinsic physical characteristics. However, we have found a strong correlation between US signal intensity and some clinically important elements of the cartilage. Moreover, the present study revealed that ultrasound can detect macroscopically undetectable change in OA. Our ultimate goal is to improve the diagnostic use of arthroscopic ultrasound systems in order to detect early degeneration in human articular cartilage.

Although the current study demonstrates clinically important findings, it has some limitations. First, the number of animals and human samples is limited. In the human study, only 20 samples were evaluated, which is too small for analyzing subgroups including gender or age. Second, instead of biochemical analysis, FTIRI with univariate-based spectral analysis was used to evaluate cartilage and determine the spatial distribution of materials of interest including collagen and proteoglycan. Amide I and carbohydrate lesion values were used to measure collagen and proteoglycan, respectively, although we did not determine their actual amounts. Nowadays, it is reported that multivalent analysis provides more accurate concentration revealing subtle change [27]. In this study, even with a simple univariate data analysis, we could find the surface change in human OA cartilage. Third, the precise qualitative analysis of the type of collagen was not performed; amide I reflects the total amount of collagen but cannot distinguish its type. Type-II collagen is predominant in articular cartilage, and we obtained a strong correlation between amide I and type-II collagen in OA cartilage in the pretest [28]. Therefore, we believe the data mainly represent changes in type-II collagen, but the data for amide I involves type-I and other minor collagen types. Finally, our current method of ultrasound evaluation could not be performed from the outside of the skin. We believe that our method is less- invasive because of the availability during arthroscopic surgery using non-contact probe, however this methods is not non-invasive. In parallel with the further accumulation of the basic information, we think it important to improve the method so as to evaluate and quantitate the cartilage from the skin surface.

In conclusion, we showed the ability of ultrasound to distinguish microscopic OA change in both animal and human cartilage, especially reflecting the information of the degeneration of superficial zone. We believe that the ultrasound is a potential method for diagnosing early subtle changes in OA at the preclinical stage.
